# Longitudinal pilot study of oxygen saturation indices in healthy preterm infants

**DOI:** 10.1038/s41390-023-02741-9

**Published:** 2023-08-04

**Authors:** Olivia Falconer, Savannah Ivy, Dana Le Carpentier, Johanna Gavlak, Natasha Liddle, Emily Senior, Paula Lowe, Philippa Crowley, Aneurin Young, Mark J. Johnson, Robert Mark Beattie, Hazel J. Evans

**Affiliations:** 1 Department of Neonatal Medicine, University Hospital Southampton NHS Foundation Trust, Southampton, UK; 2 University of Southampton School of Medicine, Southampton, UK; 3 Department of Child Health, University Hospital Southampton NHS Foundation Trust, Southampton, UK; 4 NIHR Southampton Biomedical Research Centre, University of Southampton and University Hospital Southampton NHS Foundation Trust, Southampton, UK; 5 Department of Paediatric Gastroenterology, University Hospital Southampton NHS Foundation Trust, Southampton, UK; 6 Department of Paediatric Respiratory Medicine, University Hospital Southampton NHS Foundation Trust, Southampton, UK

## Abstract

**Background:**

This study aimed to determine patterns of nocturnal pulse oximetry indices in moderate to late preterm infants, and investigate the relationship between oxygen desaturations, the apnoea hypopnoea index, and both corrected gestational and postnatal age.

**Methods:**

21 healthy infants born at 32 + 0 - 36 + 6 weeks gestation underwent serial nocturnal pulse oximetry studies and respiratory polygraphy studies until 40 weeks corrected gestational age (CGA). The main outcome measures were number of >3% oxygen desaturations/hour (ODI3), mean oxygen saturations, and number of apnoeas and hypopnoeas/hour.

**Results:**

Median ODI3 increased between weeks 1 and 3 from 49.9 to 85.4/hour (*p* = 0.017). Mean oxygen saturations reached a corresponding nadir of 96.0% in week 3, then increased to 96.8% in week 6 (*p* = 0.019). Mixed effects modelling demonstrated that ODI3 and mean saturations were influenced by postnatal age but not CGA (*p* < 0.05). Desaturations frequently occurred without an apnoea or hypopnoea.

**Conclusion:**

ODI3 rises then falls during the first 8 weeks of life in moderate to late preterm infants, independently of CGA. These interesting preliminary results highlight the importance of further serial data collection to generate age-specific normal ranges, and develop a better understanding of respiratory control in preterm infants.

**Impact:**

- The frequency of *>*3% oxygen desaturations (ODI3) in healthy moderate to late preterm infants rises then falls after birth, peaking in postnatal week 3. There is a corresponding nadir in mean saturations.
- There were significant non-linear relationships between ODI3/mean saturations and postnatal age, but not corrected gestational age.
- The majority of brief oxygen desaturations occurred without an apnoea or hypopnoea.
- Normal ranges for oxygen saturation indices are not known in this population. These results demonstrate the need for further serial data collection to generate age-specific normal ranges and inform oxygen prescribing guidelines.

## Introduction

Nocturnal pulse oximetry studies are increasingly used in preterm infants to determine whether there is a need for supplemental oxygen on discharge from the neonatal unit.^1^ Recently efforts to determine the optimal oxygen saturation range to target in preterm infants, particularly those born before 28 weeks gestation, have improved our understanding of the risks and benefits of oxygen therapy in this population. The debate has focused on balancing the risks of complications of extreme prematurity, including retinopathy of prematurity, necrotising enterocolitis, bronchopulmonary dysplasia and pulmonary hypertension. However, few studies have included infants born between 32 + 0 and 36 + 6 weeks gestation, who constitute 84.5% of the preterm population.^2^ Although most are not discharged with supplemental oxygen, at term equivalent age they experience more frequent episodes of apnoea and intermittent hypoxaemia than term neonates.^3,4^ The consequences are not well understood, but sustained apnoeas lasting ≥20 s are associated with neurodevelopmental impairment in preterm infants,^5^ and intermittent hypoxia during the first week of life restricts growth in rodent models.^6^ Moderate to late preterm infants have poorer growth and neurodevelopmental outcomes^7–10^ compared to infants born at term,^11^ so understanding the patterns and underlying mechanisms of brief desaturations is important.

Nocturnal pulse oximetry produces a continuous recording of overnight oxygen saturations, which is analysed to give summary indices including mean saturations, oxygen desaturation indices (number of times per hour the oxygen saturation falls by >3% and >4%, or ODI3 and ODI4 respectively), and percentage time spent with saturations below various thresholds during sleep. To inform clinical decision making, these indices need to be compared to age-specific normal ranges. These exist for term infants, but there are insufficient data for preterm infants.^1^ Data from older studies may not be applicable to current practice because of changes to pulse oximeters over the last two decades. Newer oximeters have shorter 2 s averaging times, increasing the detection of brief desaturations.^12^ They also utilise artefact rejection algorithms to remove motion artefact, an important consideration in infants and young children who move regularly during sleep. This leads to higher recorded mean saturations compared to older models.^13,14^

The aetiology of desaturations can be identified using respiratory polygraphy (RP). Respiratory effort and airflow are measured, and the resulting trace is analysed to give the number of apnoeas and hypopnoeas per hour (apnoea hypopnoea indices). Events are categorised as central, obstructive or mixed, depending on whether they result from a lack of respiratory drive from the brain, from respiratory effort in the presence of airway obstruction, or a sequential combination of the two. Sighs, or spontaneous large breaths of at least twice the amplitude of the preceding 10 breaths, occur frequently in preterm infants and are often followed by a pause in respiratory effort and airflow.^15,16^ If associated with a ≥3% desaturation, this is defined as a sigh-central apnoea, but if not it is sometimes referred to as a sigh-pause.^16^ The relative frequency of these two event types is a measure of the vulnerability to desaturation following a respiratory disturbance. Periodic breathing is another form of respiratory instability commonly seen in early infancy, characterised by a succession of central pauses lasting at least 3 s each, separated by no more than 20 s of normal breathing.^17^

The primary aim of this study was to determine the practicality of using overnight oximetry to make serial measurements of oxygen saturation indices in healthy preterm infants born between 32 + 0 and 36 + 6 weeks, from birth to 40 weeks corrected gestational age (CGA). Secondary aims were to investigate the aetiology of desaturations using RP, to determine whether infants’ vulnerability to oxygen desaturation with apnoeic episodes changes over time, and to assess the acceptability of home RP studies in this group. We hypothesized that the number of desaturations would gradually fall with increasing CGA, and that the majority of desaturations would occur following central apnoeas.

## Methods

### Subjects

Participants were recruited from the Neonatal Intensive Care Unit at University Hospital Southampton between October and December 2019, and between September 2020 and January 2021. Eligible participants were those born between 32 + 0 and 36 + 6 weeks gestation, with no known respiratory or cardiac conditions, or conditions predisposing to sleep disordered breathing at the time of recruitment. Infants were required to be off respiratory support or supplemental oxygen at the time of study entry. Infants whose parents were unable to understand a verbal explanation of the study in English, and families in which there were safeguarding concerns were excluded. Informed consent was obtained from participants’ parents.

### Measurements

Relevant demographic and clinical characteristics were collected from electronic patient records. Infants underwent nocturnal pulse oximetry studies either in the neonatal unit or at home if they had been discharged. Post-ductal saturations were measured continuously overnight between approximately 7 pm and 7 am, using an adhesive probe on the foot, and recorded using a Rad-8 (Masimo, CA) pulse oximeter with a 2 s averaging time and a 2 s resolution. The sensitivity and specificity of Masimo oximeters for detection of oxygen desaturations have been reported as 98–99% in children and infants.^18,19^ Nursing staff or parents were asked to complete a sleep log to ensure accurate identification of sleep periods. Recordings were analysed using VisiDownload software (Stowood Scientific, UK), which reports whole number desaturations greater than 3% or 4% (ODI3 and ODI4) i.e desaturations of at least 4.0% and 5.0% respectively. Outcomes were mean saturations, ODI3, and percentage time with saturations below 94%, 92% and 90%.

Respiratory polygraphy recordings were obtained using a SomnoTouch device and analysed using Domino Light software (Somnomedics, Germany). Events were scored by sleep physiologists experienced in scoring infant studies, according to AASM (American Academy of Sleep Medicine) criteria for respiratory events in children.^17^ Outcomes were the frequencies of central, obstructive and mixed apnoeas and hypopnoeas, and of sigh-pauses and sigh-central apnoeas, as well as the presence or absence of periodic breathing.

### Protocol

This was a prospective observational study in which infants underwent nocturnal pulse oximetry at entry, then once every completed week of CGA + /−2 days until 40 weeks CGA. Respiratory polygraphy was performed at the same time as pulse oximetry at study entry, 36 and 40 weeks CGA. Recordings were excluded if they had <4 h sleep artefact free recording time. Studies did not go ahead if participants were considered to be unwell by the clinical team and/or parents. Missed or failed studies were repeated where possible. Studies with ODI3 > 100 and mean saturations <94% were repeated within 4 days, and a clinical review of the participant was carried out. If the participant was found to be clinically well, the original studies were included in the analysis but repeat studies were excluded to avoid introducing bias. Alarms on the study oximeters were switched off, but for infants on the neonatal unit, sleep study monitoring was carried out alongside standard pulse oximetry monitoring, with saturations of ≥91% considered acceptable.

### Data analysis

Sample size was pragmatic based on the expected number of deliveries during the recruitment period, as the primary aim was to determine the practicality of making serial measurements of overnight oxygen saturations. Outcome measures were grouped by week of postnatal age (PNA) or CGA when the sleep study was done, or by the presence or absence of periodic breathing. Missing data were omitted from the analysis, so groups contained both independent and repeated measures from individual infants. Differences between groups were investigated using the Wilcoxon signed rank test. Trends observed in the oximetry data were tested using mixed effects modelling using the lme4 package^20^ for R.^21^ For mixed effects models, to account for repeated measures in individual infants, participant identity was treated as a random intercept. To test for any significant influence of postnatal age in days, CGA and sex on ODI3 and mean saturations, models were formed using progressively greater orthogonal polynomials of these variables. Models were then compared using the likelihood-ratio test, with a *p* < 0.05 considered significant. Where there was no significant difference between models, the simpler was selected.

## Results

### Study population

**Fig. 1:**
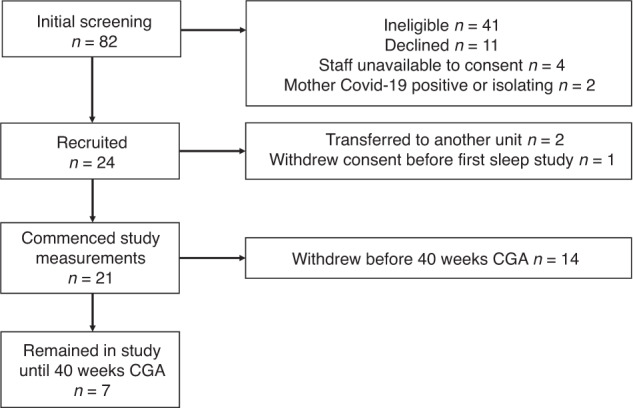
Screening and recruitment flowchart. *n* = number.

Parents of 35 eligible infants were approached, 24 consented to take part, and 21 infants had sleep studies (Fig. 1). Only one infant required non-invasive respiratory support for >72 h, and all infants were off oxygen therapy by 53 h of age (Table 1). One infant received invasive ventilation and surfactant at delivery, and was extubated on admission to the neonatal unit <1 h later. Three infants received caffeine, of whom two stopped treatment three days before the first pulse oximetry recording, and one stopped four days before the second recording. None underwent RP within 7 days after their last dose of caffeine. In total 91 oximetry and 24 RP studies were suitable for analysis. A further 17 oximetry and 22 RP studies were excluded or omitted due to inadequate sleep time, the parent declining, the baby being unwell, or technical failure. A randomly selected sample of 4 studies (from 4 different participants) were scored separately by 2 physiologists, and 100% agreement was found. Five oximetry studies met criteria for repeat because of having ODI3 > 100 and mean saturations <94%, but no infants commenced supplemental oxygen or respiratory support during the study period. Seven infants (33%) participated in oximetry recordings until 40 weeks CGA, and five infants (24%) underwent their final RP study at 40 weeks CGA, although 76% participated until at least 38 weeks CGA. The mean sleep duration of included studies was 439 min (standard deviation (SD) 117 min) for oximetry, and 414 min (SD 93 min) for RP.

**Table 1:** (a) Characteristics of study cohort. 1(b) Number (percent) of participants who received respiratory support and therapies, and duration of treatment. Some individuals received more than one type of support/therapy. 1(c) Timing of first and final nocturnal pulse oximetry and respiratory polygraphy measurements, given as median (IQR).

a)			
Characteristic		*n* (%)	Mean (SD)
Sex:	Female	7 (33.3)	
	Male	14 (66.7)	
Gestational age at birth (weeks)			33.6 (0.8)
Birth weight (kg)			2.20 (0.51)
Birth weight centile			52 (34)
Type of delivery:	Spontaneous vaginal	8 (38.0)	
	Instrumental	5 (23.8)	
	Caesarean section	8 (38.0)	
Type of milk during admission:	Breast only	2 (9.5)	
	Artificial formula only	4 (19.0)	
	Both	15 (71.4)	
Haemoglobin at age < 24 h			179 (17.7)
CGA at discharge (weeks)			36.2 (0.8)
Duration of admission (days)			18 (6.5)

b)		
Type of support/therapy	*n* (%)	Median (IQR) duration (hours)
Any respiratory support	11 (52.4)	30 (8–54)
Supplemental oxygen	6 (28.6)	3 (0–5.5)
Low-flow oxygen	0	0
High-flow	10 (47.6)	7.5 (5.3–46.8)
CPAP	5 (23.8)	14 (9–25)
Invasive ventilation	1 (4.8)	1 (1–1)
Surfactant	1 (4.8)	N/A
Caffeine within 7 days of study period	3 (14.3)	N/A

c)			
		Nocturnal pulse oximetry	Respiratory polygraphy
Postnatal age (days)	First	5 (3–9)	8 (4–14.5)
	Final	36 (25–43)	15 (8.5–40)
Corrected gestational age (weeks)	First	33.6 (33.0–34.4)	35.3 (33.8–36.1)
	Final	38.6 (37.0–40.0)	36.3 (35.1–39.9)

*SD* standard deviation, *IQR* interquartile range, *CPAP* continuous positive airway pressure.

### Oximetry data

**Fig. 2:**
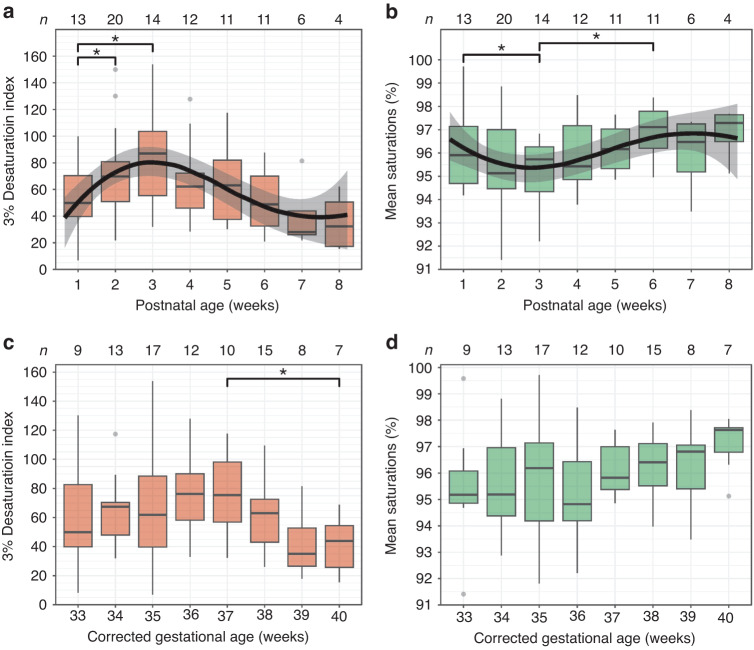
Plots showing variation in oximetry indices with postnatal age and corrected gestational age. Boxplots show 3% oxygen desaturation index (ODI3) (**a**, **c**) and mean saturations (**b**, **d**) in each week of postnatal age (**a**, **b**) and corrected gestational age (**c**, **d**). ‘Box’ represents interquartile range (IQR), with ‘whiskers’ extending no further than 1.5*IQR from either end of the ‘box’. Grey dots are data points that lie beyond this range. All data were included in statistical analysis. *n* = number of participants in each group. *p* < 0.05 for pairs of groups indicated by brackets. Black lines in (**a**) and (**b**) illustrate the fixed effect portion of the optimised mixed effects regression models, with grey shaded areas illustrating the 95% confidence interval.

When infants were grouped by PNA, ODI3 increased significantly between weeks 1 and 2 (*p* = 0.047) and weeks 1 and 3 (*p* = 0.017), and peaked at 85.4 events per hour in week 3 (Table 2, Fig. 2a). Correspondingly, mean saturations decreased from week 1 to 3 (*p* = 0.049), then increased to 96.8% in week 6 (*p* = 0.019) (Fig. 2b). Infants spent a median of 5.5% of sleep time with saturations below 92% in postnatal week 2, and this fell to 1.1% in week 7 (*p* = 0.031). Percent time with SpO_2_ below 90% and 94% also decreased between the same time points (*p* = 0.031). At study entry, there were no differences between infants who had received respiratory support and those who had not, for ODI3 (median 50.38 vs 57.8, *p* = 0.53) or mean saturations (median 95.05 vs 95.96, *p* = 0.48).

**Table 2:** Nocturnal pulse oximetry indices in infants grouped by (a) postnatal age (PNA) in weeks +/− 2 days at the time of recording, with week 1 beginning at birth; and (b) completed weeks of corrected gestational age (CGA) +/− 2 days.

a)						
PNA (weeks)	*n*	ODI3	Mean SpO_2_	% time SpO_2_ < 90%	% time SpO_2_ < 92%	% time SpO_2_ < 94%
1	13	49.9 (39.7–70.4)	95.9 (94.7–97.1)	1.8 (0.5–3.7)	5.5 (2.0–9.2)	13.8 (6.2–25.7)
2	20	69.8 (50.9–80.9)	95.1 (94.5–97.0)	3.0 (1.3–5.6)	5.5 (2.1–10.8)	14.4 (6.3–29.9)
3	14	85.4 (52.6–100.4)	96.0 (94.4–96.5)	2.7 (1.5–8.7)	5.3 (3.3–13.3)	15.1 (8.5–25.4)
4	12	62.2 (43.3–72.3)	95.4 (94.9–96.6)	2.0 (1.3–6.5)	4.6 (2.6–9.5)	10.7 (6.1–21.5)
5	11	65.8 (36.6–86.0)	95.9 (95.3–97.1)	2.8 (0.8–3.6)	4.7 (1.9–6.0)	9.6 (5.5–14.1)
6	11	53.1 (36.6–71.2)	96.8 (96.1–97.8)	1.8 (0.7–3.0)	3.5 (1.2–4.6)	7.3 (2.7–8.9)
7	6	27.1 (22.9–39.9)	96.9 (95.5–97.3)	0.5 (0.4–0.7)	1.1 (0.8–1.5)	2.7 (1.9–6.7)
8	4	32.3 (17.3–50.6)	97.3 (96.5–97.6)	0.9 (0.1–1.8)	1.7 (0.5–3.3)	7.0 (5.4–9.8)

b)						
CGA (weeks)	*n*	ODI3	Mean SpO_2_	% time SpO_2_ < 90%	% time SpO_2_ < 92%	% time SpO_2_ < 94%
33	9	49.9 (39.9–82.7)	95.2 (94.9–96.1)	1.1 (0.5–3.0)	5.5 (2.0–6.6)	13.8 (10.4–20.7)
34	13	67.5 (48.0–70.4)	95.2 (94.4–97.0)	1.8 (1.1–4.1)	5.1 (2.2–9.2)	16.8 (4.4–26.1)
35	17	61.9 (39.7–88.5)	96.2 (94.2–97.1)	2.1 (0.8–7.3)	3.8 (1.9–15.8)	8.5 (5.6–34.1)
36	12	76.3 (58.2–90.1)	94.8 (94.2–96.4)	3.0 (1.6–5.8)	6.6 (3.2–13.4)	17.2 (8.9–33.3)
37	10	75.6 (56.9–98.0)	95.8 (95.4–97.0)	3.2 (1.9–5.3)	5.9 (3.4–8.2)	11.7 (8.4–14.9)
38	15	63.1 (43.1–72.7)	96.4 (95.5–97.1)	1.8 (1.3–3.1)	3.6 (2.6–5.9)	7.4 (4.7–14.1)
39	8	35.1 (26.5–52.8)	96.8 (95.4–97.1)	0.6 (0.5–3.6)	1.4 (1.1–6.5)	8.1 (2.5–13.6)
40	7	43.9 (25.7–54.4)	97.6 (96.8–97.7)	0.9 (0.5–1.9)	1.4 (1.0–3.6)	4.5 (2.7–7.0)

Data are presented as median (interquartile range).

*n* number of oximetry studies, ODI3 3% oxygen desaturation index.

When grouped by CGA, ODI3 was stable from 33 to 37 weeks, then decreased to median 43.9 events per hour at 40 weeks (*p* = 0.031) (Fig. 2c). There were no significant differences in mean saturations between gestational age groups (Fig. 2d). Percent time with SpO_2_ < 92% was higher at 36 and 37 weeks (median 6.6 and 5.9) compared to 40 weeks (1.4, *p* < 0.032). Similarly, percent time with SpO_2_ < 90% was higher at 36 and 37 weeks (median 3.0 and 3.2) than at 40 weeks (0.9, *p* < 0.032). Percent time <94% fell between 34 and 40 weeks (median 16.8 vs 4.5, *p* = 0.047), and between 36 and 38 weeks (17.2 vs 7.4, *p* = 0.042).

The 5 studies requiring repeat occurred when infants were aged 2–4 weeks PNA and at 33–36 weeks CGA. Clinical review of the infants confirmed them to be well, and none required further investigation or treatment. One repeat study failed and one could not be repeated within the required timeframe. The remaining 3 studies showed lower ODI3 than the originals, and two showed higher mean saturations.

### Mixed effects modelling

Mixed effects modelling was used to adjust for repeated sleep studies performed on individual infants. Figure 2 shows a non-linear relationship between PNA and ODI3, and between PNA and mean saturations. This was confirmed by forming models using polynomials of postnatal age to account for these non-linear relationships. After model optimisation, there was found to be a significant non-linear relationship between ODI3/mean saturations and postnatal age (Table 3). Models were not improved by the inclusion of CGA and sex, indicating that the statistically significant relationship between ODI3/mean saturations and postnatal age is not influenced by these factors. Figure 2 plots the fixed effects portion of the optimised models as a trendline overlaid on the boxplots, demonstrating a good visual match with the relationship seen in the boxplots. The model becomes less precise after 7 weeks of life, as shown by the wider 95% confidence interval.

**Table 3:** Effect sizes and 95% CI (confidence intervals) for fixed effects of optimised mixed effects models of the relationship between postnatal age and (a) ODI3 (3% desaturation index) and (b) mean oxygen saturations.

a)	Effect on ODI3 (95% CI)	*p* value
Postnatal age (days)	−69 (−116 to −22)	0.015
Postnatal age (days): second orthogonal polynomial	−94 (−141 to −48)	<0.001
Postnatal age (days): third orthogonal polynomial	90 (44 to 135)	<0.001

b)	Effect on mean saturations (95% CI)	*p* value
Postnatal age (days)	3.0 (0.3 to 5.6)	0.030
Postnatal age (days): second orthogonal polynomial	2.7 (0.1 to 5.3)	0.061
Postnatal age (days): third orthogonal polynomial	−3.1 (−5.7 to −0.5)	0.020

### Respiratory polygraphy data

The majority of events were central (68.0%), 10.0% were obstructive, 1.5% were mixed and 20.7% were undefined. Median central apnoea hypopnoea index (CAHI) showed a similar rising and falling pattern to ODI3 with advancing PNA, peaking at postnatal week 2, though this did not reach statistical significance. When grouped according to CGA, median CAHI was highest at 36 weeks (Table 4). The median number of respiratory events per hour across all studies was 24.4, much lower than the overall median ODI3 of 62.0. Sigh-pause and sigh-central apnoea frequency remained constant across the study period, with an overall median frequency of 2.7 and 1.0 per hour respectively. Periodic breathing was present in 12 recordings (57%), and was not significantly associated with postnatal age, CGA, sex, ODI3, mean saturations, time with SpO_2_ < 92%, CAHI or the proportion of sighs that were followed by a central apnoea.

**Table 4:** Respiratory polygraphy data table. AHI = apnoea hypopnoea index, or number of apnoeas and hypopnoeas per hour. Numbers shown are median (interquartile range).

	Study entry (33–35 weeks)	36 weeks	40 weeks
*n*	9	10	5
Central AHI	6.5 (5.2–16.4)	19.6 (9.1–25.7)	5.2 (3.2–21.1)
Obstructive AHI	2.0 (0.8–2.4)	3.6 (2.4–6.3)	0.7 (0.0–1.4)
Mixed AHI	0 (0–0.2)	0.3 (0–0.7)	0 (0–0)
Undefined AHI	8.8 (6.6–9.9)	4.6 (0.7–9.3)	1.9 (0.4–5.2)
Sigh-pause index	2.9 (2.7–4.3)	2.3 (2.0–3.0)	3.2 (1.0–3.4)
Sigh-central apnoea index	0.9 (0.4–2.3)	1.0 (0.8–1.4)	1.9 (0.7–3.0)
Proportion of post-sigh events that were followed by a desaturation	0.3 (0.1–0.5)	0.3 (0.2–0.5)	0.4 (0.2–0.5)

## Discussion

This study has generated preliminary results from a small number of longitudinal measurements of oxygen saturation indices, using new generation oximeters in moderate to late preterm infants both in the hospital and home setting. We have shown that ODI3 rises and then falls over the first 8 weeks of life, peaking in week 3, in infants born at 32–35 weeks gestation. Mean saturations mirror this pattern, reaching a corresponding nadir in week 3. Mixed effects modelling shows that there are significant non-linear relationships between ODI3/mean saturations and postnatal age, but not CGA. Changes in the frequency of central apnoeas and hypopnoeas between birth and 40 weeks CGA may contribute to these patterns in oximetry indices. However, infants’ vulnerability to desaturation following an apnoea (indicated by the proportion of post-sigh events that were followed by a desaturation) did not change during this period. Oxygen desaturations were much more frequent than apnoeas and hypopnoeas, suggesting that there are additional mechanisms driving desaturations.

There were no differences in ODI3 or mean saturations at study entry for infants who had received respiratory support compared to those who had not. One infant received invasive ventilation, and another received >72 h non-invasive ventilation, and neither of these were outliers for ODI3 or mean saturations at weeks 1 or 3. Three infants received caffeine within 7 days of study entry. The half-life of caffeine in preterm infants is approximately 86 h, so blood levels may remain therapeutic for approximately 7 days after the last dose.^22–24^ Unfortunately none of the infants who received caffeine, all of whom were born at 32 weeks gestation, underwent RP at study entry, and only one had RP at 36 weeks. Therefore it is not possible to determine whether caffeine had any independent effect on apnoeas in this study.

Direct comparison with previous studies is limited by inconsistencies in the definitions of desaturation and apnoea in the literature, and differences between pulse oximeters.^13,25,26^ To our knowledge there have been no previous longitudinal studies of nocturnal pulse oximetry alongside RP in healthy moderate and late preterm infants. One recent oximetry study in infants born at 34–36 weeks gestation reported an increase in ODI3 from birth to week 2, then a decrease over the following two weeks. There was no difference between infants born at different gestational ages.^3^ Our study has shown a similar pattern in a cohort that includes infants born at 32–33 weeks, and found corresponding patterns in other measures including mean saturations. Cross-sectional measurements of mean saturations from recent studies of infants born at 32–36 weeks are similar to our results.^3,27^ Our study has added to the findings of previous studies by using longitudinal measurements to characterise the non-linear trend in mean saturations in the first 8 weeks of life. A study including term infants found a lower ODI3 (least squares mean 29.3, 95% CI 23.5–36.6, *n* = 34) on day 2–3 after birth^3^ than the preterm infants in our study had at 40 weeks CGA. This suggests that the frequency of intermittent desaturations may remain higher in preterm infants at 40 weeks CGA than in infants born at term, and further studies comparing these two groups directly would be valuable.

RP measurements from a study of term infants aged 1 month showed a predominance of central events,^28^ and found central and obstructive apnoea hypopnoea indices that were very similar to our results in preterm infants at 40 weeks CGA. Our study shows that central events are the predominant type of event throughout the period of study. Though the small number of RP measurements has not allowed for detailed analysis, central apnoeas do appear to rise and fall similarly to ODI3 in the first few weeks of life, whereas other apnoea types do not. This suggests that central events play an important role in causing desaturations.

We have shown that episodes of desaturation are much more common than apnoeas and hypopnoeas in preterm infants during sleep. This suggests that desaturations occur as a result of additional mechanisms. Preterm neonates are more susceptible to ventilation perfusion mismatching and intrapulmonary shunting of deoxygenated blood, as a result of their small size and immature lungs.^29^ Distal airway collapse due to end-expiratory volumes below closing volumes, sudden distal airway and pulmonary vascular constriction due to airway hypoxia, and the resulting accelerated blood flow through the lungs may all reduce the diffusion of oxygen into the blood leading to hypoxaemia.^29^ These mechanisms could increase preterm infants’ vulnerability to intermittent desaturation in the absence of changes in the respiratory pattern. These mechanisms alone however, would not explain why episodes of desaturation increase after birth. We speculate that rapidly increasing metabolic demand associated with increasing energy expenditure and nutrition after birth might contribute to the increase in desaturations.^30^ Modelling of oxygen desaturation in preterm infants shows that decreases in saturations are accelerated by higher metabolic consumption of oxygen.^31^ A fall in overall Hb and HbF concentration in the first few weeks after birth may also be important, since a lower HbF concentration would bring about a greater drop in oxygen saturations for a given decrease in the partial pressure of oxygen in the tissue.

The results of this study should be interpreted cautiously in view of the small number of participants, and more data are required to generate normative ranges for use in clinical practice. No infants born at gestations beyond 35 + 0 were eligible for recruitment, as very few infants born at 35 and 36 weeks gestation without comorbidities require admission to the Neonatal Intensive Care Unit. The group of infants recruited is therefore more homogeneous, but future studies aiming to include later gestational ages will need to adapt the recruitment strategy. Relatively few sleep studies were carried out at 7 and 8 weeks PNA, because the majority of infants completed their final sleep study before reaching 7 weeks of age. This was either because they had already reached 40 weeks CGA or because they were withdrawn early. The estimated size of the effect of PNA on ODI3 and mean saturations is therefore less precise at 7 and 8 weeks PNA, as indicated by wider confidence intervals (Fig. 2). Serial RP measurements proved challenging to complete at home in this population in the context of the Covid-19 pandemic. More than half of the RP studies that were omitted were due to take place after discharge home, and the majority of these were during the pandemic, when staff were able to offer limited technical support to parents. The remainder were predominantly at study entry, when parents often expressed concern that the equipment was too much for their baby to wear. For similar reasons, although periods of sleep and wake are more accurately determined by amplitude-integrated electroencephalogram (EEG), instead the sleep log and features of the trace were used. Home polysomnography (including EEG) is increasingly being undertaken in children^32^ but was not considered practical in this study of preterm infants, some of whom were discharged home during the study and underwent assessment at home. Excluding non-English speaking families is another potential limitation, but for this preliminary study, it was not possible to provide parents with documents and technical support in other languages. No large studies of nocturnal pulse oximetry alongside RP in preterm infants have previously been carried out, and the longitudinal patterns in oximetry indices seen in this study add important information to existing data from other small studies. The oximetry data presented have been used to estimate the sample size required for a larger study. While the quantity of RP data is small, the predominance of central events, together with the finding that many more desaturations occurred than scorable apnoeas is of interest and warrants further investigation.

The time points for measurements were chosen according to the hypothesis that outcomes would depend mainly on CGA. The small number and uneven spread of RP measurements across the study period meant that analysis by week of PNA was not possible. Some respiratory events were classified as undefined, when paradoxical breathing or low signal from the effort bands made it impossible to distinguish central from obstructive hypopnoeas. Measuring nasal pressure flow or thermistor flow can mitigate this, but was not done because many parents found the nasal prongs too intrusive.

## Conclusion

This pilot study demonstrates important changes in oxygen saturation measures in healthy moderate to late preterm infants over the first few weeks of life, in particular that the number of desaturation events increases and mean saturations fall over the first 3 weeks of postnatal life. These results highlight the importance of further serial data collection in this age group to generate age-specific normal ranges for use in clinical practice. Our preliminary results indicate that such data would be likely to influence oxygen prescribing guidelines. In addition, the timing of sleep studies to determine oxygen requirements for discharge home will require careful consideration and discussion between clinicians and families. Further exploration of the mechanisms behind these patterns is important to increase our understanding of the causes and consequences of oxygen desaturation in preterm infants.

## Data Availability

The datasets generated during and analysed during the current study are available from the corresponding author on reasonable request.
